# Supplementary material for: Exploring the influence of different habitats and their volatile chemistry in modulating sand fly population structure in a leishmaniasis endemic foci, Kenya
Source: PLoS Negl Trop Dis. 2021 Feb 1;15(2):e0009062. doi: 10.1371/journal.pntd.0009062 (PMC7877749; doi:10.1371/journal.pntd.0009062)
Supplement: Attachment — Submitted filename: Response to Review comments Sept302020.docx [file pntd.0009062.s001.docx]

30^th^ September 2020.

Editor-in-Chief,

PLoS Neglected Tropical Diseases,

Shaden Kamhawi (PhD).

Dear Prof Kamhawi,

**Re: Manuscript re-submission: PNTD-D-20-01096**

We are pleased to resubmit the manuscript entitled **"Exploring the influence of different habitats and their volatile chemistry in modulating sand fly population structure in a leishmaniasis endemic foci, Kenya**" for further consideration for publication in your journal. We have made all the changes you suggested in your letter and addressed all the comments of the reviewers in the notes below. Please note that the reviewers’ comments are shown in bold type and our responses in plain type italics.

Yours sincerely,

David P. Tchouassi

Thank you very much for submitting your manuscript "Exploring the influence of different habitats and their volatile chemistry in modulating sand fly population structure in a leishmaniasis endemic foci, Kenya" for consideration at PLOS Neglected Tropical Diseases. As with all papers reviewed by the journal, your manuscript was reviewed by members of the editorial board and by several independent reviewers. In light of the reviews (below this email), we would like to invite the resubmission of a significantly-revised version that takes into account the reviewers' comments.
 
We cannot make any decision about publication until we have seen the revised manuscript and your response to the reviewers' comments. Your revised manuscript is also likely to be sent to reviewers for further evaluation.
 
When you are ready to resubmit, please upload the following:

[1] A letter containing a detailed list of your responses to the review comments and a description of the changes you have made in the manuscript. Please note while forming your response, if your article is accepted, you may have the opportunity to make the peer review history publicly available. The record will include editor decision letters (with reviews) and your responses to reviewer comments. If eligible, we will contact you to opt in or out.

[2] Two versions of the revised manuscript: one with either highlights or tracked changes denoting where the text has been changed; the other a clean version (uploaded as the manuscript file).

Important additional instructions are given below your reviewer comments.

Please prepare and submit your revised manuscript within 60 days. If you anticipate any delay, please let us know the expected resubmission date by replying to this email. Please note that revised manuscripts received after the 60-day due date may require evaluation and peer review similar to newly submitted manuscripts.
 
Thank you again for your submission. We hope that our editorial process has been constructive so far, and we welcome your feedback at any time. Please don't hesitate to contact us if you have any questions or comments.
 
Sincerely,

Geraldine Marie Foster, PhD
Guest Editor
PLOS Neglected Tropical Diseases

Helen Price
Deputy Editor
PLOS Neglected Tropical Diseases

***********************

**Reviewer's Responses to Questions**

<b>Key Review Criteria Required for Acceptance?</b></br></br>
As you describe the new analyses required for acceptance, please consider the following:</br></br>

<b>Methods</b></br></br>
-Are the objectives of the study clearly articulated with a clear testable hypothesis stated?</br>
-Is the study design appropriate to address the stated objectives?</br>
-Is the population clearly described and appropriate for the hypothesis being tested?</br>
-Is the sample size sufficient to ensure adequate power to address the hypothesis being tested?</br>
-Were correct statistical analysis used to support conclusions?</br>
-Are there concerns about ethical or regulatory requirements being met?</br></br>

**Reviewer #1: Because representative substrates were used to characterize the VOCs from the animal shed and house, it is difficult to assess the relationship between the sand fly epidemiologic data and the habitat-specific VOC profiles without more information:**

**1. For the animal dung used as substrate for the animal shed VOCs: was the dung collected from the same animal sheds and during the same time periods where the sand fly collections took place? If so, please clarify this in the text. If not, please explain why this did not occur. If you have information about the animal species from which the dung originated, please also include this information.**

*Re: Further clarification has been included in the manuscript text to address these concerns. Cow dung volatiles were collected from selected animal sheds were traps were set. The same applies to volatiles from humans and termite mounds. Thus, we have volatile data from only a subset of habitat types sampled for sand flies. We have included the following sentences in the study design section to improve on clarity. 1) Odor collection was conducted concurrently during sand fly trapping in January 2020 (L203-204). 2) The human volunteers and dung were from represented houses and animal sheds, where sand flies were trapped (L209-210). Livestock has been replaced with cow (addressed throughout manuscript).*

**2. For the human-worn socks used as a substrate for VOC detection: were these socks worn by the same humans living in the houses where sand fly collection took place, and during the same time periods? If so, please clarify this in the text. If not, please explain why this did not occur.**

*Re:* *Kindly refer to our response to query 1 above.*

**3. The data does not convey the inter-individual variability in VOC profiles (both in humans and from the animal dung). Please show data that makes explicit which VOCs were specific to a minority of samples from one habitat type, versus VOCs which were present in the majority or all the samples from one habitat. This is important to assess how representative or generalizable the VOC profile is for a given habitat.**

*Re: We reckon that inter-individual differences in volatile profiles is a common occurrence when assessing profile differences in different substrates even for same species. Our aim was to explore the qualitative volatile profile differences between the different substrates, and this was feasible given the replicate trials per substrate type (3-4) (L 216). Thus, no detailed quantitative comparison was made given that quantities of substate from which volatiles were trapped, were not standardized.*

**4. Why were VOCs not directly captured from the air in the animal shed and house habitats? The authors showed they could successfully capture odorants directly from the termite mounds. Direct capture of VOCs from the environment would help mitigate concerns about the generalizability of the VOC data gathered from proxy substrates (as raised in points 1 through 3 above). If there are intractable technical obstacles to such an experiment, please discuss these considerations in the text.**

*Re: Our design included negative controls: cleaned unused socks (human) and air or blank from the environment (dung and termite mound) (L216-218). No additional control from air inside houses was conducted. The field pump utilized ensures only purified air from the environment was passed over the substrate thereby minimizing any potential contribution to the volatile profile of the target substrate. Thus, the ensuing volatile profiles reflects the content of the treatment. The volatile profile for humans agrees with those of previous findings established using similar protocol (See Tchouassi et al 2019; Olaide et al 2019). Nonetheless, we fully agree with the suggestion to* *capture the contribution of background volatiles especially in houses indoors which has been included as a shortcoming of the study in the discussion (L501-509).*

**Reviewer #2: -Are the objectives of the study clearly articulated with a clear testable hypothesis stated?
This can be improved see my attached comments**

-Is the study design appropriate to address the stated objectives?
There are some problems in this area, see attached comments.

-Is the population clearly described and appropriate for the hypothesis being tested?
Not applicable.

-Is the sample size sufficient to ensure adequate power to address the hypothesis being tested?
Not entirely clear – see comments.

-Were correct statistical analysis used to support conclusions?
See comments

-Are there concerns about ethical or regulatory requirements being met?
No

--------------------

<b>Results</b></br></br>
-Does the analysis presented match the analysis plan?</br>
-Are the results clearly and completely presented?</br>
-Are the figures (Tables, Images) of sufficient quality for clarity?</br></br>

**Reviewer #1: - Please discuss in the text possible reasons for why total sand fly abundance was higher in the January 2020 collection than December 2018 (Table 2).
- Lines 304-305: “Among the species, seasonality based on trapping period only influenced catches of P. saevus and S. antennata (Table 2).” I don’t think “seasonality” is the appropriate term here, since January and December are very close to each other in the calendar. Please clarify this language.**

*Re: The concern regarding seasonality has been considered and the sentence revised and now reads: Among the species, variation in abundance based on the trapping period was evident for P. saevus and S. antennata (L338-339). Perhaps, a variation in habitat microclimatic factors such as temperature, moisture and organic matter known to influence sand fly abundance (Wijerathna & Gunathilaka, 2020) may have contributed to the differential abundance trends observed between the two trapping periods.*

*This has been included in the discussion. L432-435….*

**Reviewer #2: -Does the analysis presented match the analysis plan?
Not applicable

-Are the results clearly and completely presented?
Nearly

-Are the figures (Tables, Images) of sufficient quality for clarity?
yes**

--------------------

<b>Conclusions</b></br></br>
-Are the conclusions supported by the data presented?</br>
-Are the limitations of analysis clearly described?</br>
-Do the authors discuss how these data can be helpful to advance our understanding of the topic under study?</br>
-Is public health relevance addressed?</br></br>

**Reviewer #1: - Lines 462-463: The authors state that, “the current study provides empirical evidence of possible involvement of volatiles in habitat selection,” however the data provided do not fully support this conclusion, as currently phrased. This argument is further weakened by the use of proxy substrates for the habitat VOCs, as discussed above. To support this claim, the authors need to show an association between the habitat-specific abundance of different sand fly species and habitat-specific VOCs or VOC profiles. In the absence of such data, the authors can state that this is a limitation of the study, and that determining an association between the sand fly species abundance and VOC profiles reported in this study remains to be performed.**

*Re: We have acknowledged the fact that our experimental design did not allow for determining an association between the sand fly captures and volatile profiles from the different habitats. This concern has been considered favorably and included as a limitation of the present study. L501-509.*

**Reviewer #2: please see comments**

--------------------

<b>Editorial and Data Presentation Modifications?</b></br><br/>
Use  this section for editorial suggestions as well as relatively minor modifications of existing data that would enhance clarity. If the only modifications needed are minor and/or editorial, you may wish to recommend “Minor Revision” or “Accept”.

**Reviewer #1: Other than the modifications discussed above, no other modifications needed.**

*Re: No response needed*

**Reviewer #2: in some areas explanations and grammar can be improved. many of these are minor issues but would help improve the overall presentation of the paper.**

--------------------

<b>Summary and General Comments</b></br></br>
Use  this section to provide overall comments, discuss strengths/weaknesses of the study, novelty, significance, general execution and scholarship. You may also include additional comments for the author, including concerns about dual publication, research ethics, or publication ethics. If requesting major revision, please articulate the new experiments that are needed.

**Reviewer #1: REVIEW SUMMARY
Hassaballa et al. report the results of a cross-sectional survey of sand fly species abundance and diversity in Rabai village of Baringo County, Kenya, an endemic area for both visceral and cutaneous leishmaniasis. The study focuses on comparing sand fly species and diversity in three different kinds of habitats in the village – animal sheds, termite mounds, and houses. The authors hypothesize that sand fly habitat selection is due in part to different profiles of volatile organic compounds (VOCs) that act as odorants in the different habitats. The authors use LC-MS to catalogue VOCs directly from the termite mounds, and from proxy substrates of the animal sheds and houses, namely animal dung and human-worn socks, respectively. Overall, the study presents useful data for sand fly epidemiology and leishmania vector control efforts in East Africa, however minor revisions are necessary before I can recommend the manuscript for publication, as detailed in my critiques in the above sections.**

*Re: This is noted. Thank you.*

**Reviewer #2: This is an interesting study combining field observations with laboratory analysis. The study addresses important considerations in relation to east Africa vectors of Leishmaniasis. This type of work is severely lacking and therefore this work address major gaps in our knowledge.

My main concern is that the background and methodology of the work needs further explanation and clarification. The results also require clarification and the conclusions and Discussion should clearly reflect the limitations of the study.

However I do not want the authors to feel that this reviewer does not appreciate the efforts that they have gone to. Field work in this area is difficult and any contribution is valuable. With some small effort they can make this a very good and useful paper.**


**specific comments**

L2 Sand fly
*Corrected L2*

**L12 Missing author affiliations**
*Corrected*. *L7-8*

**L35 “guilds" = Communities?**
*Corrected to composition L38*

**L41-43 What does this mean – a greater number of species present in animal sheds?**

*Re:* *We have included the following sentence to discuss the implication of this finding: This finding indicates broader attractiveness of both sexes of sand flies and females of varying physiological states to animal sheds potentially used as breeding or resting sites but also as signal for host presence for a blood meal. L46-48).*

**L44-46 This is not clear Human houses = socks? Animal sheds =dung? Termite mounds =????**

*Re: The sentence has been revised and now reads…...human foot odor on worn socks (houses indoors), livestock dung (animal sheds) and termite mounds (enclosed vent), revealed a total of 47 volatile organic compounds. L50-53*

**L51-53 It is not clear how this conclusion is arrived at. It would be acceptable (and more accurate) is you replace “show” with “suggest” in this sentence.**

*Re: This has been revised following the suggestion. L58*

**L69 understanding the ecology of the vector is essential.**

*Re: The sentence has been revised to capture this suggestion: Understanding the ecology of sand flies is critical to developing control measures L65.*

**L78 Did you formally try to associate species abundance or numbers with volatile abundance or diversity.
I mean in houses or animal shelters the increased sand fly species diversity and abundance might be related to the greater quantity of compounds present in  those collecting sites compared to the termite mounds. Also, those sites are likely to present a greater number of other sand fly relevant resources compared to the termite mounds. Why choose termite mounds?**

*Re: We have acknowledged the fact that our experimental design did not allow for determining an association between the sand fly captures and volatile profiles from the different habitats Kindly refer to our response to similar comment raised by reviewer 1 above and that this shortcoming of the study has been addressed in the discussion (L501-509). The choice for examining the selected habitats including termites was guided by previous studies in the study site, used as resting habitats of epidemiologic importance for sand fly species. This information is indicated in the introduction. L130-132.*

**L106 It is not clear what you mean here. Is vector control used against leishmania vectors in Kenya or not? I think you need to examine the Kenyan Ministry of Health policy in this area and report it here.**

*Re: This section has been revised to improve clarity and now reads:* *In Kenya, leishmaniasis control largely focuses on case management with minimal sand fly control mainly in response to outbreaks. The increasing public health impact of leishmaniasis calls for the need to develop integrated vector management (IVM) strategies for prevention and control of sand flies. L99-103.*

**L108 Give an example and a reference**

*Re: A reference was provided L99-103*

**L110 “ In response to leishmaniasis outbreaks, spraying of houses with insecticides is common … “ This is contradicting the statement in L106 so this whole section needs to be clarified.**

*Re: Please refer to our response to the query above (L99-103)*

**L111 “Thus, integrated vector management strategies for prevention and control of the sand fly vectors of the disease remain limited”  Vague and not consistent with previous statements.**

*Re: Kindly see our response above Line 99-103*

**L113 This is not clear –reduction of human transmission is the objective of vector control.
L113 "… which can be achieved through improved understanding of sand fly behavioral adaptations”  I’m not sure that t this is a logical consequence of the first part of the sentence.**

*Re: The sentence has been revised and now reads…New ways of controlling leishmaniasis can be achieved through improved understanding of sand* *fly behavioral ecology, specifically resting and breeding habitats. L106-108.*

**L114 Are you talking about the situation in Kenya or East Africa or more generally?**

**L115 “…insecticides or other biocontrol agents …” I think there is room (and need) to be specific – give examples**.

*Re: The section on sand fly control targeting breeding and resting sites has been reviewed in the introduction and provided examples not only in East Africa but elsewhere including Brazil and Asia (L106-124). Metarhizium anisopliae has been included as an example of a biological control agent (L110).*

**L119 Which ecological trait?**

*Re: This has been changed to ecological sites. L114.*

**L120-122 The authors use 2 citations (both are cited incorrectly) to support this point, unfortunately neither of the citations support the statement. Perhaps they suggested this option in the discussion? In any case the authors need to very carefully check the citations and make sure that actually support the points being made. The authors need to distinguish between actual (i.e. real control methods performed on a routine basis by recognised agencies and proposed or experimental control methodologies.**

*Re: We have reviewed the section on sand fly control targeting breeding and resting sites in the introduction and provided examples not only in East Africa but elsewhere including Brazil and Asia (L106-124) and including appropriate citations (refs 13, 20, 21). We have qualified methods based on vector control or experimental research.*

**L113-129 The section on control is very weak. It needs to be rewritten there are plenty of good examples from India/Bangladesh, Iran and South America particularly Brazil.**

*Re: We have reviewed this section of the manuscript and included other valuable literature describing work on vector control, environmental management and biocontrol agents targeting suitable resting/breeding sites for sand flies (L106-124).*

**L141 The sentence structure is not clear – are you talking about 2 foci? Or one focus of leishmaniasis?**

*Re: Revised to read ‘leishmaniasis focus’. L126, 128.*

**L141 “..guilds..” It would be helpful to define what this term means in the context of this work.**

*Re: This word has been deleted and changed to composition. Line 135*

**L142-143 What is the basis for this hypothesis? You have not linked sand fly distribution with different odour previously in the introduction therefore you need to have a section before you define the objective of the study that sets out the logical argument for conducting this work.**

*Re: This hypothesis has been revised and now reads: In this study, we tested the hypothesis that the composition of sand flies varies in different habitats and that the habitats differ in their volatile chemistry that could influence sand fly selection. (L135-138). A justification for this link was provided in the ensuing sentences after the hypothesis.*

**L146-148 This is an association study and therefore you cannot truly say that distribution is related to odour. This can only be done by a manipulative experiment. Please recognise this in the Discussion.**

*Re: We have revised the hypothesis to reflect this concern and now reads.In this study, we tested the hypothesis that the composition of sand flies varies in different habitats and that the habitats differ in their volatile chemistry that could influence sand fly selection. (L135-138).*

**L157 Balanites spp..** *Done (L153)*

**L170 use “handheld GPS device” as it is better grammatically.** *Done (L164, 179)*

**L179 How were the sites selected? This is an important consideration and you need to elaborate the point – also how many of each?**

*Re: The habitats are interspersed in the study sites and information that traps were positioned at 50 - 100 m intervals along a transect was provided. L175-181. Only one trap was set in each habitat type at a given time.*

**L180-181 Poor expression**

**L181 How many traps in each trapping site?**

*Re: Clarification on the trapping effort for each habitat has been clarified and included in the study design section viz: The number of trap night were 36, 10 and 34 for termite mounds, animal sheds and houses indoors, respectively, in December 2018. Trap nights in January 2020 were 22, 14 and 24 for termite mounds, animal shed and houses indoors, respectively. L181-183.*

**L181-182 Do you mean inside houses? – you should stick with your previous definition L147 of trapping sites.**

*Re: changed to houses indoors throughout the manuscript*

**L187-189 Why not store them in ethanol?**

*Re: Liquid N2 works fine as well, as we intended to perform other analysis on the captured samples after identification*

**L197 Is there something missing here? does gum chloral clear the preparation if so how are they fixed on the glass side?**
*Re: The sentence has been clarified to include…..mounted using Berlese’s medium (which contains gum Arabic and chloral hydrate). (L 195)*

**L206. This is a substantially different way of collecting the volatiles. And one immediate consequence that I can think of is that you would be likely to collect less volatiles from the termite mounds compared to the other 2 odour sources. I wonder why you did not use a standard so that you can directly compare the entrainments. Please add this point to your Discussion as well as commentary on the weaknesses in the methodology.**

*Re: We acknowledge this methodological challenge to efficiently sample volatiles from termite mound and recognize this could be improved by incorporating other volatile trapping methods. This has been discussed (L501-509)*

**L 207 How many?**

*Three-four replicates per habitat substrate. L216*

**L209 details of solvent and oven temperature.**

*Re: Dichloromethane (DCM) as the solvent used was indicated (L219, 229) including the oven temperature L232-234)*

**L229 delete “to”**

*Done L230*

**L235 replace “was” with were obtained**

*Done L236*

**L239 Confirmation would require comparison of retention time on 2 columns of differing polarity. You need to moderate your claim of “confirmed structures. Tentatively identified by comparing with library Data only.**

*Re: We have revised the sentence and now reads…Where available, compounds were identified by co-injection with authentic standards. We have also included a description on compound identification based on retention indices (L240-255).*

**L254 for “treatment” do you mean location?**

*Re: Treatment has been changed to habitat type. L267-268 and else where in the manuscript*

**L260 this is a Discussion point or could be included in the Introduction as a justification for collecting Sergentomyia.**

*Re: This information was captured in L430-432 in the discussion and so deleted from the data analysis section.*

**L267 this is not clear please explain and rephrase**

*Re: Revised as suggested. L240-241*

**L278 I am assuming verbal? – please confirm.**

*Re: This has been clarified and changed to verbal consent. L291*

**L289 P.** *Re: Corrected L302*

**L302 this is an interesting word which implies that is was caught elsewhere in your study. I think it’s worth giving the details.
L303 “…trapping season …” I don’t think you can describe these collection time points as different “trapping seasons”. One collection time point is December the other in January – these are in the same season even though separate in time. It would have been interesting to collect in a different season. You actually refer to the season “dry season” later in the manuscript.**

*Re: We used the term trapping session or period not season. L316*

**L314 “…than in houses indoor …” replace with, inside houses and in other places where you use this style.**

*Re: This has been considered and changed accordingly. L313-322 and changed throughout the manuscript (houses indoors)* **L380 Table 4 this table doesn’t tell me how many compounds that were present in each habitat were not identified. Some of the “identifications” are really quite suspect. I’m not really sure why you need to try and identify all the compounds. You can note absence/presence, quantities and total numbers of compounds and base your comparison of these sites on that data (however my other points about standards is also relevant.
L384 It is not clear if this is the retention index of the standard or the compound in the entrained extract. I would expect to see both in this table (as well as on a different column).**

*Re: Identification of compounds in our substrates was established using the GC-MS by comparison with reference databases and further, where available by co-injection with authentic standards. Our aim was to explore the qualitative volatile profile differences between the different substrates, and this was feasible given the replicate trial per substrate type (3-4). Thus, no detailed quantitative comparison was made given that quantities of substate from which volatiles were trapped, were not standardized. Significant information can be conveyed from knowledge of identified compounds thus our decision to focus on this than unidentified ones. Nonetheless, we have reviewed the content of Table 4 and excluded o-cymene from the list. Consequently, a revised Fig 6 is included.*

*Information on the retention indices of the compounds in the entrained extract is indicated. The following information has been included in the manuscript text in the study design: Identification of the VOCs in each substrate was achieved based on their retention indices (RI). RI was determined with reference to a homologous series of normal alkanes C_8_-C_23_ and calculated using the equation below as described by Van den Dool and Kratz (Van den Dool and Kratz et al 1963) and comparison with published literature (Karioti et al 2004; Babushok et al 2011; Njuguna et al 2018).*

***RIx=100 n_0_ + 100 (R_T_x – R_T_n_1_) / (R_T_n_1_-R_T_n_0_)***

*With:*

*x = the name of the target compound*

*n_1_ = n-alkane C_n1_H_2n1+2_ directly eluting before x*

*n_0_ = n-alkane C_no_H_2nO+2_ directly eluting after x*

*R_T_ = retention time*

*RI = retention index*

*Line 244-255*

**L391-405 This is all good**

*Re: Many thanks.*

**L408 I think you need to qualify this by indicating collections made in Kenya (or East Africa) because it is not necessarily the case in other regions of the world.
L414-416 this seems a key point that the Phlebotomus spp are found at all 3 sites. However it’s not entirely clear that that conclusion is supported by the evidence. The numbers are very low and it’s not clear from the Methods how much trapping effort was deployed to achieve these catches. Was it one night or 100 nights?**

*Re: Clarification has been made on the trapping effort realized for each habitat (L181-183). To control for this disparity trapping effort per habitat, data on sand fly abundance per trap for each habitat was subjected to analysis and providing a standard measure to allow for comparisons. For instance, despite fewer replicates in animal sheds, this habitat type had overall higher abundance and diversity of sand flies. Thus, we feel that the observed abundance patterns may reflect each habitat effect.*

**L435 “… like also found …” can this be expressed in a better way?**

*Re: Corrected…Expression replaced with ‘similarly’ L450*

**L453 Unfortunately I am not entirely convinced by this analysis. You could have tried some very simple manipulative experiments that would have relied on odor alone thereby vastly strengthening your conclusion. However as long as you clearly indicate that you understand the limitations of the study and discuss them the results have some validity. In my view you have tentatively identified some compounds that may act as attractants for the sand fly species in question. You have not shown that abundance and species distribution is linked to those compounds.**

*Re:* *We have acknowledged the fact that our experimental design did not allow for determining an association between the sand fly captures and volatile profiles from the different habitats. This concern has been addressed above with the following information included in the discussion as a limitation of the study:* ***‘****Additional studies to establish a clear association of sand fly catches with specific volatile profiles from the examined substrates will be helpful (L506-518).*
