# Supplementary material for: Exploring the influence of different habitats and their volatile chemistry in modulating sand fly population structure in a leishmaniasis endemic foci, Kenya
Source: PLoS Negl Trop Dis. 2021 Feb 1;15(2):e0009062. doi: 10.1371/journal.pntd.0009062 (PMC7877749; doi:10.1371/journal.pntd.0009062)
Supplement: Attachment — Submitted filename: Response to reviewer comment Nov252020.docx [file pntd.0009062.s002.docx]

25^th^ November 2020.

Editor-in-Chief,

PLoS Neglected Tropical Diseases,

Shaden Kamhawi (PhD).

Dear Prof Kamhawi,

**Re: Manuscript re-submission: PNTD-D-20-01096**

We are pleased to resubmit the manuscript entitled **"Exploring the influence of different habitats and their volatile chemistry in modulating sand fly population structure in a leishmaniasis endemic foci, Kenya**" for further consideration for publication in your journal. We have made all the changes you suggested in your letter and addressed the comment of Reviewer 2 in the notes below. Please note that our response is indicated in bold type italics below the reviewer’s comment.

Yours sincerely,

David P. Tchouassi

Dear Dr. Tchouassi,
 
Thank you very much for submitting your revised manuscript "Exploring the influence of different habitats and their volatile chemistry in modulating sand fly population structure in a leishmaniasis endemic foci, Kenya" for consideration at PLOS Neglected Tropical Diseases. As with all papers reviewed by the journal, your manuscript was reviewed by members of the editorial board and by several independent reviewers. The reviewers appreciated the attention to an important topic. Based on the reviews, we are likely to accept this manuscript for publication, providing that you modify the manuscript according to the review recommendations.
 
As noted below, there remains one outstanding point for clarification regarding the identification of compounds on one column using co-injection with standards. All other requested revisions have been successfully addressed.
 
Please prepare and submit your revised manuscript within 30 days. If you anticipate any delay, please let us know the expected resubmission date by replying to this email.  

When you are ready to resubmit, please upload the following:

[1] A letter containing a detailed list of your responses to all review comments, and a description of the changes you have made in the manuscript. 
Please note while forming your response, if your article is accepted, you may have the opportunity to make the peer review history publicly available. The record will include editor decision letters (with reviews) and your responses to reviewer comments. If eligible, we will contact you to opt in or out

[2] Two versions of the revised manuscript: one with either highlights or tracked changes denoting where the text has been changed; the other a clean version (uploaded as the manuscript file).

Important additional instructions are given below your reviewer comments.

Thank you again for your submission to our journal. We hope that our editorial process has been constructive so far, and we welcome your feedback at any time. Please don't hesitate to contact us if you have any questions or comments.
 
Sincerely,

Geraldine Marie Foster, PhD
Guest Editor
PLOS Neglected Tropical Diseases

Helen Price
Deputy Editor
PLOS Neglected Tropical Diseases

***********************


Reviewer's Responses to Questions

<b>Key Review Criteria Required for Acceptance?</b></br></br>
As you describe the new analyses required for acceptance, please consider the following:</br></br>

<b>Methods</b></br></br>
-Are the objectives of the study clearly articulated with a clear testable hypothesis stated?</br>
-Is the study design appropriate to address the stated objectives?</br>
-Is the population clearly described and appropriate for the hypothesis being tested?</br>
-Is the sample size sufficient to ensure adequate power to address the hypothesis being tested?</br>
-Were correct statistical analysis used to support conclusions?</br>
-Are there concerns about ethical or regulatory requirements being met?</br></br>

Reviewer #1: All critiques were addressed in revision, there are no methodological concerns.

Reviewer #2: (No Response)

--------------------

<b>Results</b></br></br>
-Does the analysis presented match the analysis plan?</br>
-Are the results clearly and completely presented?</br>
-Are the figures (Tables, Images) of sufficient quality for clarity?</br></br>

Reviewer #1: All critiques were addressed in revision, there are no concerns regarding the results.

Reviewer #2: (No Response)

--------------------

<b>Conclusions</b></br></br>
-Are the conclusions supported by the data presented?</br>
-Are the limitations of analysis clearly described?</br>
-Do the authors discuss how these data can be helpful to advance our understanding of the topic under study?</br>
-Is public health relevance addressed?</br></br>

Reviewer #1: The conclusions have been appropriately revised to reflect the experimental findings -- there are no further concerns.

Reviewer #2: (No Response)

--------------------

<b>Editorial and Data Presentation Modifications?</b></br><br/>
Use  this section for editorial suggestions as well as relatively minor modifications of existing data that would enhance clarity. If the only modifications needed are minor and/or editorial, you may wish to recommend “Minor Revision” or “Accept”.

Reviewer #1: Minor grammatical revisions will be needed before final publication.

Reviewer #2: (No Response)

--------------------

<b>Summary and General Comments</b></br></br>
Use  this section to provide overall comments, discuss strengths/weaknesses of the study, novelty, significance, general execution and scholarship. You may also include additional comments for the author, including concerns about dual publication, research ethics, or publication ethics. If requesting major revision, please articulate the new experiments that are needed.

Reviewer #1: This is a revised manuscript by Hassaballa et al. reporting the results of a cross-sectional survey of sand fly species abundance and diversity in Rabai village of Baringo County, Kenya, as well as cataloguing profiles of volatile organic compounds (VOCs) in different habitats within the village. The characterization of VOCs as part of sand fly epidemiological studies is a novel approach and has the potential to be impactful for vector control efforts in East Africa. The authors have adequately addressed all the critiques I raised in my review. I commend the authors for this research, and recommend the revised manuscript be accepted for publication.

Reviewer #2: The authors have addressed the majority of the concerns that i raised major and minor. I apologies for confusing sessions with seasons and am happy with the meaning of both. My only outstanding issue is that you still can't categorically claim to identify compounds on one column by co-injection with standards.It doesn't detract from the manuscript/paper if they authors are clear about this issue and explain what they have done. But it will matter to those who read the paper and see that the authors are trying to claim full identification of compounds when they have not achieved that. However, this is a technical point and perhaps to most readers not important. But I will leave it to the authors to address this issue and "tone down" the identification rhetoric!

Still a nice piece of work well worthy of publication.

***Re: Thank you. We have revised the information relating to identification of the compounds in the methods section and now reads…‘Where available, the identities of VOCs were confirmed by co-injection and comparison of mass spectral data with authentic standards. Retention indices (RI) were determined with reference to a homologous series of normal alkanes C_8_-C_23……_L226-228)***

PLOS authors have the option to publish the peer review history of their article (<a href="<https://journals.plos.org/plosntds/s/editorial-and-peer-review-process#loc-peer-review-history>" target="_blank">what does this mean?</a>). If published, this will include your full peer review and any attached files.<br><br>

If you choose “no”, your identity will remain anonymous but your review may still be made public.<br><br>

<b>Do you want your identity to be public for this peer review?</b> For information about this choice, including consent withdrawal, please see our <a href="<https://www.plos.org/privacy-policy>" target="_blank">Privacy Policy</a>.

Reviewer #1: Yes: Joshua R. Lacsina

Reviewer #2: No
